# A randomised feasibility trial of VR-enhanced cue exposure therapy versus neutral VR exposure for cocaine use disorder, with wearable monitoring and JITA-EMA: Feasibility randomised controlled trial protocol

**DOI:** 10.1371/journal.pone.0358193

**Published:** 2026-10-30

**Authors:** Eileen Brobbin, Matteo Cella, Alexandre Copello, Simon Coulton, Hannah Darvell, Jerome Di Pietro, Colin Drummond, Joe Duffy, Amos Folarin, Steven Glautier, Tayler Holborn, Ceyda Kiyak, Jazmin Molai, Thomas Phillips, Heet Sankesara, Daniel Stahl, Callum Stewart, Lucia Valmaggia, Colin Williams, Paolo Deluca

**Affiliations:** 1 National Addiction Centre, Institute of Psychiatry, Psychology & Neuroscience, King’s College London, London, United Kingdom; 2 Department of Psychology, Institute of Psychiatry, Psychology & Neuroscience, King’s College London, London, United Kingdom; 3 School of Psychology, University of Birmingham, Birmingham, United Kingdom; 4 Centre for Health Service Studies, University of Kent, Canterbury, United Kingdom; 5 Department of Biostatistics and Health Informatics, Institute of Psychiatry, Psychology & Neuroscience, King’s College London, London, United Kingdom; 6 Department of Psychology, University of Southampton, Southampton, United Kingdom; 7 Department of Psychology, University of Southern Denmark, Odense, Denmark; 8 Centre for Addiction and Mental Health Research (CAMHR), Faculty of Health Science, University of Hull, Hull, United Kingdom; 9 Centre for Youth Mental Health, University of Melbourne, Melbourne, Australia; 10 Digital XR Innovation Lab, Orygen, Parkville, Australia; Public Library of Science, UNITED KINGDOM OF GREAT BRITAIN AND NORTHERN IRELAND

## Abstract

**Background:**

Cocaine use disorder (CUD) is associated with high rates of relapse, with craving and cue reactivity playing important roles in ongoing use. Cue Exposure Therapy (CET) aims to reduce conditioned responses to drug-related cues through extinction learning, but traditional approaches may be limited by poor ecological validity. Virtual reality-enhanced Cue Exposure Therapy (VR-CET) offers a potentially more immersive and realistic method of delivering cue exposure. Concurrently, advances in wearable physiological monitoring and just-in-time adaptive ecological momentary assessment (JITA-EMA) enable the real-time assessment of craving in everyday life.

**Objective:**

To assess the feasibility of recruitment, consent, randomisation, blinding, intervention delivery and retention over 8 weeks post-randomisation.

**Method:**

This is a two-arm, parallel-group, randomised feasibility trial conducted in UK community drug treatment services. Sixty adults receiving treatment will be randomised (1:1) to either VR-CET or an active VR-Relaxation control condition. Both arms will receive six VR sessions over four weeks, alongside continuous physiological monitoring using a wearable device and smartphone-based JITA-EMA for eight weeks. Feasibility outcomes will include recruitment, retention, intervention adherence, wearable wear time, EMA completion, acceptability, and safety. Clinical outcomes will include cocaine use measured by Timeline Follow-Back, cue reactivity assessed through subjective and physiological measures, and participant-confirmed craving episodes captured via wearable-triggered JITA-EMA. Qualitative interviews with participants and service providers will explore intervention acceptability and implementation facilitators, barriers and suggestions.

**Results:**

Planned start date is July 2026, and enrolment is due to end in April 2027. Data analysis is estimated to be completed by the end of spring 2027.

**Conclusions:**

To our knowledge, this is the first feasibility RCT of VR-CET for CUD within UK drug treatment services. Results from this trial will help assess the feasibility of integrating VR-CET, continuous physiological monitoring, and real-time craving JITA within CUD treatment. This can be used to inform the design of a future definitive trial and contribute to the development of personalised, technology-enabled approaches to relapse prevention and substance treatment.

**Trial registration:** ISRCTN21409750, https://www.isrctn.com/ISRCTN21409750.

## Introduction

Current treatment for powder or crack cocaine use primarily relies on psychosocial interventions, including cognitive behavioural approaches [1,2]. However, relapse remains common, with craving playing a central role [3]. A major clinical challenge is that craving is transient, strongly context-dependent, and often triggered by cues and emotional states encountered in everyday life [4]. Craving is both a recognised diagnostic criterion in DSM-5 and ICD-11 and a predictor of relapse. Despite its clinical importance, most evidence is derived from laboratory or clinic-based assessments, limiting understanding of how craving occurs in naturalistic settings [5].

Just-in-time adaptive ecological momentary assessment (JITA-EMA), combined with wearable physiological monitoring, offers an opportunity to capture craving and cue reactivity in real time and everyday environments. By prompting assessments during periods of elevated risk, JITA-EMA can increase the likelihood of recording clinically relevant events [6]. Wearable devices can continuously measure physiological signals associated with cocaine use and craving-related arousal, including heart rate, electrodermal activity, skin temperature, and movement [7–9]. Studies have also demonstrated physiological responses during cocaine cue exposure, supporting the potential value of these measures as markers of cue reactivity [10–14].

Virtual reality-enhanced Cue Exposure Therapy (VR-CET) uses immersive environments to simulate substance-related cues and high-risk situations within a controlled setting [15,16]. The approach is grounded in classical conditioning theory [17], whereby repeated pairings of cocaine use with environmental stimuli result in conditioned responses such as craving and physiological arousal. Cue Exposure Therapy (CET) seeks to weaken these associations through repeated exposure to cues without drug reinforcement, promoting extinction learning and reducing conditioned responses [18]. VR may enhance CET by providing realistic, repeatable exposure to complex scenarios that are difficult to recreate safely in clinical settings. It also enables the rehearsal of coping strategies without access to cocaine [19] and can be tailored to individual triggers across multiple environments or contexts, potentially improving generalisation of treatment effects [20,21].

Two linked studies conducted by this research team informed the development of the present study (Ref: 24/NW/0342 and 25/ES/0061). These studies demonstrated the feasibility of combining continuous wearable monitoring with smartphone-based EMA among people receiving treatment for cocaine use disorder and provided evidence that physiological changes associated with craving can be detected in everyday life (*in preparation*). The previous VR study further refined the VR-CET cocaine scenarios, in addition to the literature review [22] and ongoing patient and public involvement (PPI). Building on this work, the current feasibility RCT will integrate VR-CET, wearable monitoring, and JITA-EMA to evaluate feasibility, acceptability, safety, and deliverability, while generating preliminary estimates of effects on cue reactivity and cocaine use outcomes to inform a future definitive trial.

The study is guided by an explicit programme theory proposing that repeated VR-CET without reinforcement will reduce cue-elicited craving and physiological reactivity through extinction learning. In parallel, wearable-triggered JITA-EMA will facilitate near-real-time identification and assessment of high-risk craving states, providing a foundation for future JITAI to support craving, relapse and recovery.

### Objectives and hypotheses

The primary objective is to assess the feasibility of recruitment, consent, randomisation, blinding, intervention delivery and retention over 8 weeks post-randomisation.

The secondary feasibility objectives are:

- To assess acceptability and adherence/data completeness for: (i) VR-enhanced CET and VR Relaxation sessions, (ii) wearable wear-time, and (iii) EMA completion.
- To obtain variance estimates of key outcomes for future sample size calculations.
- To assess the safety and tolerability of VR-enhanced CET and remote monitoring procedures (including adverse events, distress management, and cybersickness).
- To assess the feasibility and performance of the wearable-triggered JITA-EMA approach, including trigger frequency, response rates, and the proportion of triggers associated with participant-confirmed craving/high-risk states.
- To assess the feasibility and acceptability of implementation in routine services and identify barriers, facilitators, and potential solutions from the perspective of service providers and participants.

The clinical objectives are:

- Cocaine use: measured at baseline and 8-week follow-up using Timeline Follow ack (TLFB) 28. Key derived endpoints will include frequency of use, cocaine-free days, and time to first use.
- Cue reactivity: change from baseline to week 4 (end of treatment), operationalised as (i) subjective craving (e.g., Cocaine Experience Questionnaire (CEQ-S)) and (ii) physiological indices from EmbracePlus in response to a standardised static cue reactivity test.
- Craving episodes over 8 weeks post randomisation**:** frequency of participant-confirmed craving episodes captured via wearable-triggered JITA-EMA prompts, with episode severity rated on a visual analogue scale (VAS).

This study uses a feasibility RCT trial design, randomising participants into the VR-CET or VR-Relaxation arm (1:1) to investigate this.

## Methods

### Trial design

Multi-centre, individually randomised, two-arm feasibility trial with 1:1 allocation to VR-CET or VR Relaxation control arm, a 4-week intervention period (six sessions), and 8-week follow-up post-randomisation. This study has an additional service provider qualitative component. Staff working in the participating services will be invited to complete one semi-structured interview to explore service providers’ views of the intervention and implementation in routine services.

A Patient and Public Involvement (PPI) advisory board has contributed to the study design and procedures, providing input into the acceptability and feasibility of the intervention, participant-facing materials, recruitment and retention strategies, and study procedures to ensure they are appropriate and relevant to the target population.

### Participants, eligibility and settings

Six addiction clinics across two regions in England are participating sites: South London and Maudsley (SLaM) NHS drug services, NHS Humber, Waythrough, and CGL services in London and Hull.

Individuals will be able to participate if they are attending one of the participating services and meet the following criteria: 1) Treatment-seeking patients for cocaine SUD, and 2) Able and willing to provide informed consent. Individual’s will be excluded if they meet any of the exclusion criteria: 1) Evidence of severe mental illness or cognitive impairment, 2) Impaired vision or hearing that cannot be corrected (i.e., with contact lenses, glasses or hearing aid), 3) Physical inability to wear a wrist device, both arms having tattoo sleeves (as the device requires contact to ink free skin), 4) Current participation in similar research study.

Service providers: Staff at participating sites will be invited to complete a semi-structured interview to assess the feasibility and acceptability of implementing VR-CET in routine drug and alcohol services and to inform future scale-up. Inclusion criteria: 1) Health care professional working in a drug outpatient or inpatient setting in one of the participating drug services, 2) Over 18 years old, 3) Willing to provide informed consent to participate.

### Ethical approval

This study was approved by the Chelsea REC (Ref: 25/ES/0061). All participants will provide written informed consent after reviewing consent documents with research staff. Participants will be given a unique study ID that will be used to identify their data throughout the study. Only trained research staff have access to the key that matches the participant with their ID. The key is password-protected and stored on a secure server at King’s College London (KCL).

### Intervention arms

Both arms will complete six VR sessions over four weeks, wear the physiological monitor and complete daily JITA (Fig 1). The VR-CET arm will experience scenes created to elicit cocaine craving. The VR-Relaxation arm will experience scenes created to induce relaxation. The VR-Relaxation arm provides a credible control condition matched for session number, staff contact, equipment, and study procedures, allowing assessment of the feasibility and acceptability of VR-CET beyond the non-specific effects of VR participation.

**Fig 1:**
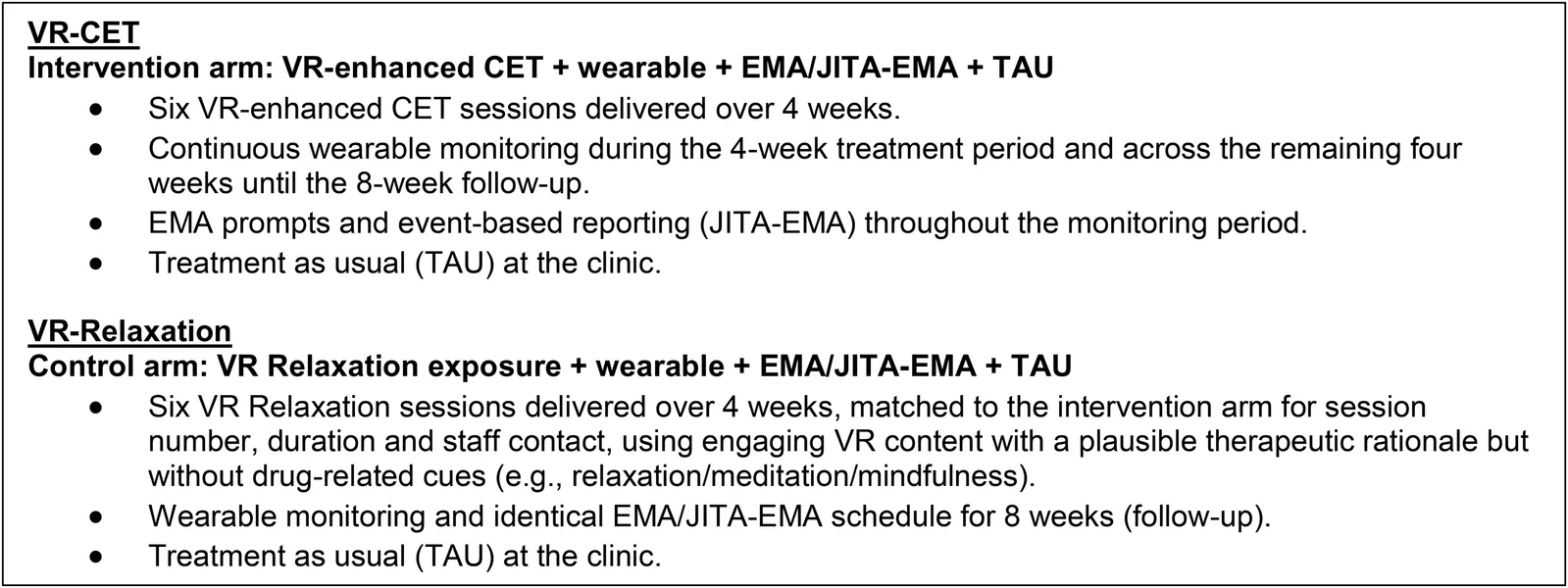
Description of VR-CET and VR-Relaxation arms.

### VR-CET

Across the six sessions, participants will complete four VR scenarios per session (24 total exposures in total). Participants will be exposed to eight pre-specified Environment × Cue combinations (Table 1). The cocaine cues designed are: Purchasing from a dealer and consuming cocaine, and observing others talk about and then use cocaine. These occur in different environments: Flat, Car, Pub, Public toilet, House party, Bathroom, Stairwell, Alleyway.

**Table 1:** Combinations of cocaine cue x environment for powder and crack.

Route of administration	Cocaine cue	Physical environment
Powder	Purchasing and consuming	Flat / Car
		Car park
		Pub / Car / Public toilet
		House party / Car / Bathroom
	Observing conversation and consumption	Flat
		Car park
		Pub / Public Toilet
		House Party / Bathroom
Crack	Purchasing and consuming	Flat / Car
		Car park
		Stairwell / Car
		Alleyway / Car
	Observing conversation and consumption	Flat
		Car park
		Stairwell
		Alleyway

After the cocaine scenarios have been completed, the researcher will guide the participant through a relaxing or distracting activity designed to de-escalate cocaine cue-elicited arousal. Participants will remain with the researcher until their self-reported craving returns to their pre-VR level. Craving will be monitored at regular intervals before participants leave the study premises. Participants will also be given a coping with craving handout to take away with them.

### VR-Relaxation

Across the six sessions, participants will complete repeated exposure to a fixed set of VR scenarios using the commercially available Liminal application [23]. The relaxation and meditation activities were chosen because they are non-drug-related, low risk, suitable for repeated use, and have a clear rationale for participants as calming or stress-management activities. They also avoid exposing participants in the control group to cocaine cues while still providing an engaging and credible VR experience. Liminal immerses users in calming virtual environments (e.g., beaches, forests, or meditation spaces) and includes guided breathing and mindfulness exercises with soothing audio-visual content. Eight pre-specified Liminal environments will be used across the six sessions, with each session comprising four scenario exposures, matching the structure and intensity of the VR-CET arm (Fig 2). The equipment used is the Meta Quest 3 headset and controllers.

**Fig 2:**
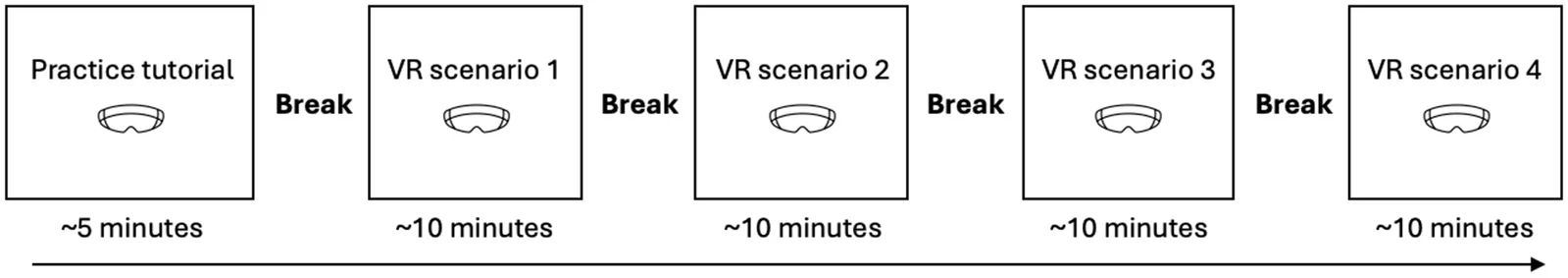
Flow diagram of a VR session.

### VR delivery

All VR sessions, research meetings and questionnaires will be delivered by a trained member of the research team. The research team have prior experience of delivering VR-CET from the previous linked study (IRAS: 357025, REC: 25/ES/0061). Prior to data collection, the team will undertake refresher training on the delivery of the VR-CET and VR relaxation procedure. Session standard operating procedures (SOPs) and distress management protocols have been developed and are in place. Distress procedures include monitoring during exposure, the option to pause/terminate the session at any time, post-session debriefing, and signposting/escalation in line with local clinical pathways.

### Procedure

Below in Figures 3 and 4, a flow chart visualises the study procedure. Participants will be recruited from the participating sites and approached either by the researcher or service staff. Staff will be aware of the study and inclusion criteria and have Participant Information Sheets (PIS) to provide. The research team will then speak to the individual directly, answer any questions and arrange a meeting if the individual meets the inclusion criteria and is willing to participate. The researcher will also attend group meetings at each service and be able to talk to service users directly and provide PIS.

**Fig 3:**
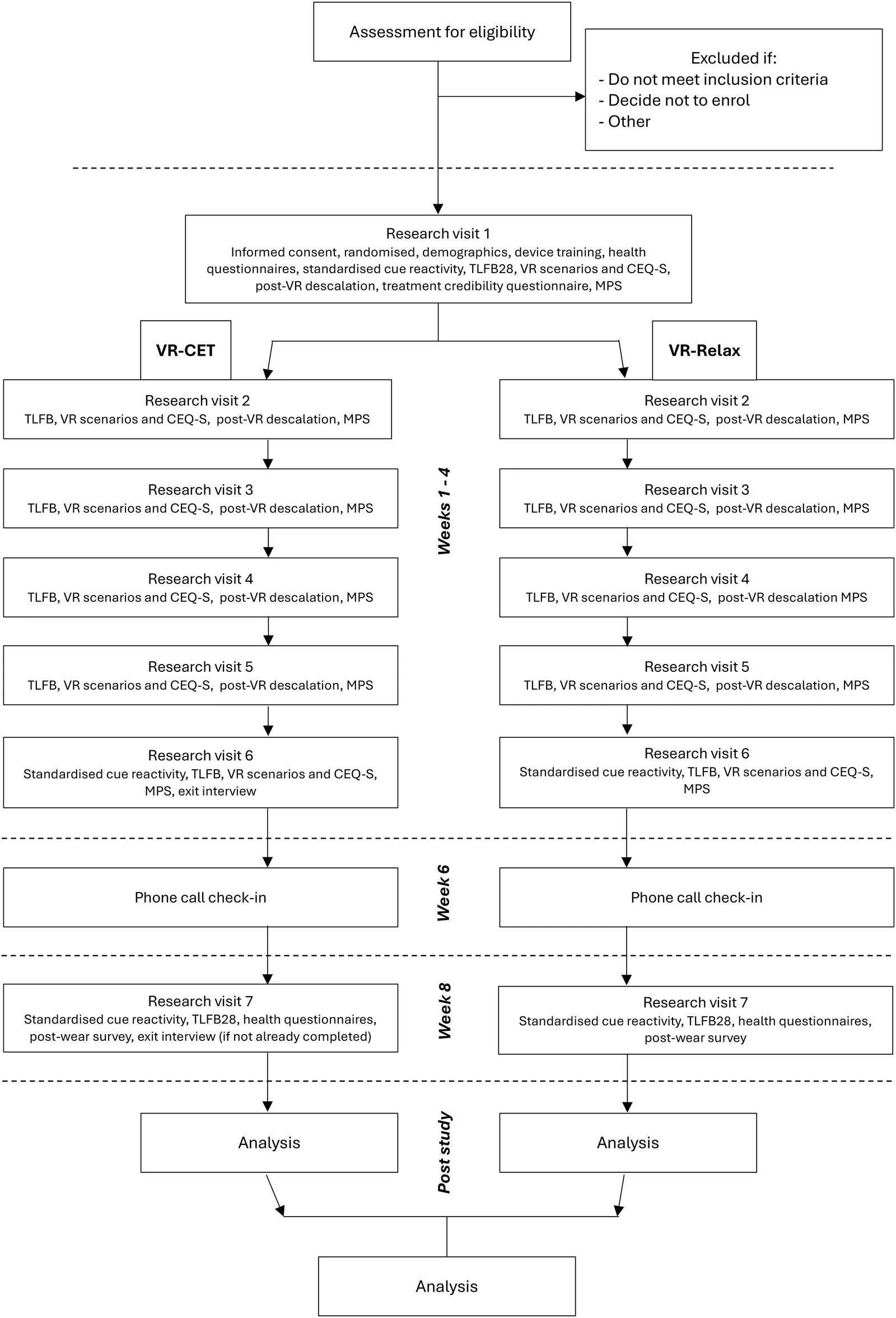
Flow chart of the study. Participants will be randomised at the first research visit. The following meetings (2–6) will be arranged for regularly over the next four weeks. A phone call check in will be completed in week 6 and the final research visit 8 weeks post-randomisation.

**Fig 4:**
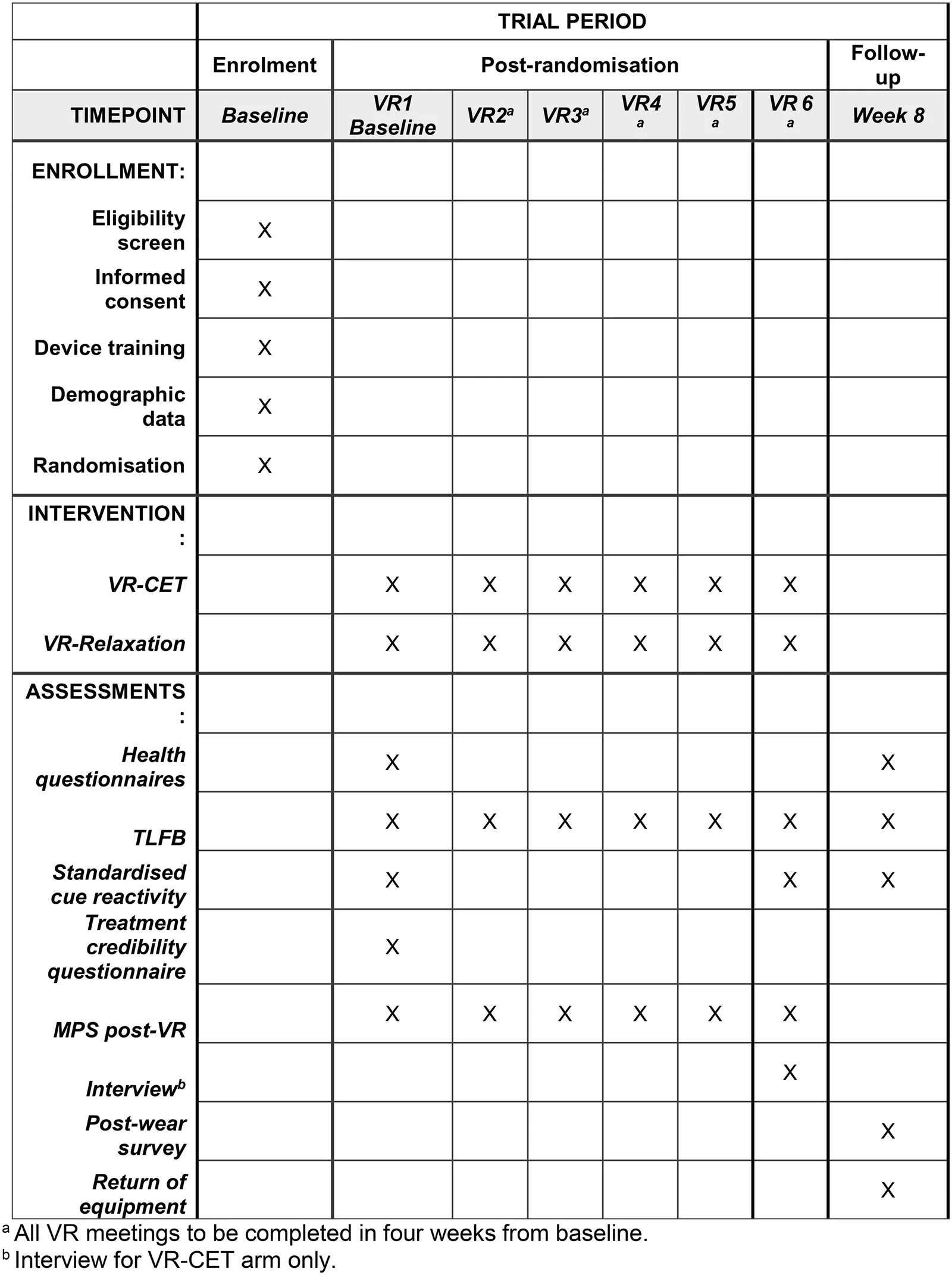
Participant timeline: Schedule of enrolment, interventions, and assessments.

Randomisation will occur in the first meeting after informed consent has been provided. The participant will be enrolled as part of the VR-CET or VR-Relaxation arm. Apart from VR content (cocaine cue exposure versus relaxation, and interview only for VR-CET arm), study procedures and meetings follow the same design. There will be 6 VR sessions arranged for within the first four weeks (intervention phase), a phone call check-in (week 6) and the final meeting (week 8). Throughout the participants will wear the Empatica and complete JITA-EMA via a smartphone application. In Fig 4 the participant timeline and schedule are shown.

Research visit 1: Participants will receive a general overview of the study and sessions, provide informed consent, be randomised, be provided with the Empatica (and trained to use it) (Figs 3 and 4), provide baseline socio-demographic data, complete health questionnaires (TAPS, Nuffield Health Questionnaire, PHQ-8, GAD-7 PSS-10) and a standardised static cue reactivity assessment and provide a Timeline Follow Back (TLFB) of the past 28 days of drug and alcohol use. Then they will complete the VR practice tutorial and the first VR session (a VR session is made up of four separate scenarios). After the four VR scenarios, and a relaxing or distracting activity (designed to de-escalate cocaine cue elicited arousal), the participants will complete a CEQ-S, treatment credibility questionnaire and Multimodal Presence Scale (MPS).

Research visits 2–5: The same VR session structure will be completed. Participants will have the option at the start of the session to complete the practical tutorial to remind themselves of how the controllers work and will complete the CEQ-S and TLFB since the previous session. After the VR scenarios a relaxing or distracting activity will be completed, followed by the MPS.

Research visit 6: Participants will complete the standardised static cue reactivity assessment followed by the same VR session procedure. Those in the VR-CET arm will complete a semi-structured interview.

Research visit 7: Participants will return the Empatica, complete a standardised static cue reactivity assessment, TLFB28, health questionnaires (TAPS, PHQ-8, GAD-7, PSS-10) and a post-wear survey. If the interview was not completed in visit 6, there will be the chance to complete the interview in this visit for the VR-CET arm.

### Service Provider

Each service provider participant will take part in an interview that will last 30–45 minutes. No follow-up interviews are planned. This interview will cover their views on the intervention and its implementation in services, guided by the Theoretical Framework of Acceptability (TFA) [24].

### Measures

#### Craving.

Craving will be assessed using three methods: (1) CEQ-S self-reported ratings collected during research visits, (2) participant-reported craving through the JITA-EMA, and (3) physiological data recorded by the Empatica EmbracePlus device.

An algorithm will be used to determine cocaine craving as measured by these physiological sensors. EMA data will be collected using the active Remote Monitoring Technology (aRMT) smartphone application, part of the Remote Assessment of Disease and Relapse (RADAR-base) platform, an open-source, GDPR-compliant system for remote data collection. JITA-EMA will be triggered by pre-specified patterns in EmbracePlus-derived physiological data, analysed in near real time using the algorithm developed in a linked study (Ref: 24/NW/0342). When a trigger threshold is reached, participants will receive a smartphone notification asking whether they are experiencing cocaine craving. Prompts will be capped at a maximum of three per day and will not be sent more than once within two hours to minimise burden; participants will have up to one hour to respond.

#### Substance use.

A TLFB [25] will be completed at the final meeting covering the past 28 days and used in the analysis to compare against the JITA-EMA data collected. The participant will be sent JITA-EMA daily and have a fixed evening EMA to report on any substance use in the past 24 hours over their 8-week participation period.

#### VR presence.

The Multimodal Presence Scale (MPS) [26] will be used to measure physical, social and self-presence of the VR scenarios.

### Outcomes

The primary feasibility outcomes are:

- Recruitment rate (e.g., number of participants referred each month and screen-to-consent ratio).
- Randomisation and intervention uptake (proportion consented who are randomised; proportion randomised who attend ≥1 VR session).
- Intervention delivery/adherence (sessions attended; proportion attending ≥4/6 sessions).
- Retention at 8-week follow-up (proportion completing follow-up).
- Approached/screened (the number of individuals approached and screened, with reasons for ineligibility/non-participation).

The secondary feasibility outcomes are:

- Acceptability of VR interventions (VR-CET and VR Relaxation).
- JITA-EMA performance: trigger frequency, response rate within 1 hour, and proportion of triggers associated with participant-confirmed craving episodes.
- Data completeness: wearable wear time, EMA completion rate, exit interview.
- Safety/tolerability: adverse events, cybersickness, distress episodes and their management.
- Implementation outcomes (service-provider perspective): barriers/facilitators and training and resource needs.
- Resource use: staff time, room/space requirements, and equipment set-up/maintenance to inform a future economic evaluation.

Primary clinical outcome

- Cocaine use: measured at baseline and 8-week follow-up using TLFB-28. Key derived endpoints will include frequency of use, cocaine-free days and time to first use.

Secondary clinical outcomes

- Cue reactivity: change from baseline to week 4 (end of treatment), operationalised as (i) subjective craving (e.g., CEQ-S) and (ii) physiological indices from EmbracePlus.
- Craving episodes over 8 weeks post randomisation**:** frequency of participant-confirmed craving episodes captured via wearable-triggered JITA-EMA prompts, with episode severity rated on a VAS.

### Sample size

This feasibility trial is not powered to test efficacy. A sample size of 60 is considered adequate for obtaining reliable feasibility parameter estimates, including robust estimates of variance of the main outcomes for future sample size calculation [27,28], and to provide adequate precision for key feasibility parameters (e.g., recruitment, retention at 8 weeks, adherence to VR sessions, wearable wear-time and EMA completion) for estimating the expected proportion with a precision of 12% and 95% confidence and to generate variance and indicative between-arm effect estimates for cue reactivity and cocaine use outcomes (i.e., frequency of use) to inform the design and sample size of a future definitive trial.

Service providers: Approximately 15 individuals. This sample size is consistent with guidance for qualitative framework analysis, where thematic saturation is typically achieved within this range for a relatively homogeneous professional group [29].

### Recruitment

To achieve adequate participant enrolment to reach the target sample size, there will be regular communication with each participating service. Research staff will attend each service regularly to inform staff of the ongoing recruitment and attend service user groups to discuss the research with eligible individuals.

Service providers: An email will be sent to all staff at participating drug services, with the PIS and research poster attached. The contact details of the research team will be included, and interested participants will be directed to contact the research team.

### Randomisation

Participants will be randomised 1:1 to the VR-enhanced CET or VR Relaxation control arm using a secure computer-generated allocation sequence using block randomisation to maintain balance between arms during recruitment (with varying block sizes to prevent prediction), stratified by site (London and Hull) and prior participation in Study 2 (yes/no). Randomisation will be performed using an online randomisation web-based system, with oversight from the trial statistician. Allocation will be generated during baseline assessment, ensuring allocation concealment, and will be accessible only to authorised staff responsible for intervention delivery.

Due to the nature of the intervention, intervention-delivery staff cannot be blinded. However, because both trial arms involve a credible VR intervention, we will aim to minimise differential expectancy by providing balanced information about both VR interventions. Statistical analyses will be conducted blinded to allocation until the primary analysis is finalised. Analyses will adjust for stratification factors where appropriate. Treatment credibility/expectancy [30] will be assessed after Session 1 to evaluate the success of this approach. Due to the small research team and the nature of the intervention, outcome assessors at the 8-week follow-up visit cannot be fully blinded to allocation. This is an acknowledged limitation; however, the primary feasibility outcomes (e.g., session attendance, retention rates) are objective and therefore less susceptible to assessor bias.

### Data collection and management

All data collection will be conducted by trained research staff in accordance with standardised operational protocols. All staff will have completed Good Clinical Practice (GCP) and protocol training before enrolment. Collected data will be entered into a secure cloud-based data spreadsheet. This allows for immediate data entry and secure data storage. Data instruments follow standardised formats to streamline assessments. Data collected is entered directly into the system. To protect participant confidentiality, all data are deidentified using numeric coding systems and stored in a password-protected database. Access to this database is restricted to a small group of authorised members, including the CI, statisticians and research staff conducting data collection. The data custodian for this study is KCL.

### Statistical analysis

#### Quantitative analysis.

The feasibility of trial procedures will be summarised using proportions and 95% confidence intervals for recruitment, consent, eligibility, treatment retention, adherence and follow-up completion. Reporting will follow the CONSORT extension for randomised pilot and feasibility trials. Analyses of clinical outcomes will focus on estimation rather than hypothesis testing, using descriptive statistics and confidence intervals to characterise the likely range of treatment effects, defined as the difference in the craving versus non-craving contrast between arms. Estimates of population variances for key outcomes will inform future sample size calculations. Patterns of missing data will be summarised descriptively. Exploratory analyses will examine whether cue reactivity indices predict subsequent cocaine use/relapse.

The JITA-EMA operational performance will also be reported (trigger frequency, response rates within 1 hour, and proportion of triggers associated with participant-confirmed craving/high-risk states). A detailed statistical analysis plan will be finalised before inspection of outcome data.

#### Qualitative analysis.

For both the service users and service provider interviews, the analysis will be directed by the TFA (Sekhon et al., 2017). Acceptability, according to the TFA, consists of 7 components: affective attitude, burden, perceived effectiveness, ethicality, intervention coherence, opportunity costs, and self-efficacy. TFA is useful for assessing the acceptability of healthcare interventions within the development, piloting and feasibility, outcome process evaluation and implementation phases of interventions (Sekhon et al., 2017). This data will be used to inform the evaluation of the acceptability and feasibility of the VR experience. The core components of TFA were used to guide the creation of the topic guide.

### Progression criteria (to a definitive trial)

Progression to a definitive, fully powered trial (or progression with modifications) will be guided by the following indicative criteria, interpreted alongside qualitative findings: (1) Recruitment: achieves a sustainable recruitment rate across sites and acceptable screen-to-consent conversion. (2) Retention: ≥ 70% complete the 8-week follow-up. (3) Intervention adherence: ≥ 70% of participants in each VR arm attend at least 4/6 sessions, with acceptable tolerability and comparable attendance across arms. (4) Credibility/expectancy: early credibility/expectancy ratings indicate both VR programmes are acceptable/credible and that marked differential expectancy is not evident. (5) Data completeness: median wearable wear-time meets the minimum threshold for analysis (≥12 h/day on ≥70% of days) and scheduled EMA completion is acceptable (≥60%). (6) JITA-EMA performance: trigger rate and response burden are acceptable (median triggers/day within a pre-specified range based on Study 1), with an adequate response rate (≥60% responded within 1 hour) and most triggers linked to participant-confirmed craving episodes. (7) Safety/tolerability: no serious adverse events (SAE) attributable to study procedures and low rates of session discontinuation due to cybersickness or distress. (8) Signal: directionally favourable and plausible estimates for reduced cue reactivity and cocaine use outcomes to justify a definitive trial.

### Interim analyses and stopping rules

No formal interim analysis or pre-specified stopping rules are planned for this feasibility trial, as it is not powered to test efficacy. In the event of unexpected safety concerns, the CI will consult the ISG and notify the REC and sponsor in accordance with the non-CTIMP safety reporting framework. Serious adverse events attributable to study procedures will be reported to the REC within 15 days of the CI becoming aware of the event.

### Safety aspects

A Data Monitoring Committee (DMC) was not required for this study because it is a small pilot study. A DMC was not required in the ethical approval process. There is an Independent Steering Group (ISG) which meets regularly to oversee the research, progression and analysis.

Previous literature reports slight irritation from wearing the Empatica, which the participant will be made aware of. There are no other expected medical complications. In the previous study (Ref: 24/NW/0342), in which the Empatica was worn for one month, two of the 72 enrolled participants reported irritation on their wrist due to the device material. All participants will be made aware that if the Empatica becomes uncomfortable, they can remove it. All participants in the previous study said that irritation reduced once the Empatica was removed for a break from wear, and no further action was needed. Another possible side effect of the VR is motion sickness and dizziness. Regular breaks have been placed between each VR scenario to minimise this risk. In the previous study, which followed a similar procedure, no motion sickness was reported by participants. Any serious adverse event occurring during the study will be reported. The research team includes supervision from a medical doctor.

### Ethical issues

This study protocol and all study documents, including the participant consent form, were approved by the Chelsea REC. Any protocol amendments will be completed appropriately, and all necessary parties will be notified.

Before being admitted to the study, participants will receive a PIS as well as a detailed explanation of the scope and possible risks of participating in a form that is understandable to them. Each participant will give consent in writing at the first research visit. There will be three copies of the consent form, one for the research team, one for the participant to keep, and one for their service records. Consent will be obtained from each participant by a member of the research team.

All participants in both the VR-CET and VR-Relaxation arms will continue with treatment as usual at their usual drug service. Both arms will wear an Empatica for the same time period and meet with the researcher the same number of times. At each research visit, the researcher will discuss how the participant is finding the study, ask if they have had any problems or concerns and answer any questions. As part of the consent form, the participant will be aware that they can withdraw from the study at any time, for any reason, without their care being affected.

The data from participants will be kept strictly confidential. Once data leaves the site, the initials and the unique identification code will only be used. The Chief Investigator (CI) declares no conflict or competing interests. The research staff completing the data collection and statisticians will have access to the full dataset.

Dissemination of the study findings is planned for publication in peer-reviewed scientific journals and presentation at international conferences.

## Results

This study has been designed to assess the feasibility of recruitment, consent, randomisation, blinding, intervention delivery and retention over 8 weeks post-randomisation. The findings from this research will contribute to the growing literature on VR-CET in substance treatment. The planned end of enrolment date is April 2027. The analysis is expected to be completed by the end of spring 2027 and to be published a year after study completion.

## Discussion

To our knowledge, this is the first trial for establishing the feasibility of VR-CET with individuals accessing treatment in UK drug services. This represents a critical translational step in the field, moving VR-CET beyond laboratory-based cue reactivity paradigms and into real-world clinical settings. The findings will therefore have direct implications for the future clinical implementation and scalability of CET-based interventions for substance use disorders.

CET has a strong theoretical basis in classical conditioning theory and aims to mitigate cravings and conditioned responses (cue reactivity) by repeatedly exposing individuals to cues while preventing substance use. Through this, conditioned responses associated with substance cues are expected to extinguish over time. Within this framework, environmental contexts, paraphernalia, social interactions, and affective states that have previously been paired with substance use acquire motivational salience and can subsequently trigger craving, drug-seeking behaviour, and relapse. A recent meta-analysis [31] indicates that CET, either as a standalone intervention or in combination with Coping or Drug Refusal Skills Training and/or Cognitive-Behavioural Therapy (CBT), demonstrates small to medium effects on outcomes such as alcohol consumption, drinking frequency, and relapse rates. Whilst encouraging, the strength of these conclusions is limited by a small evidence base and disproportionate influence of a single study employing video-based CET [32]. Consequently, although CET appears promising, further evidence is required before widespread clinical implementation can be recommended.

Importantly, while immersive technologies such as VR have increasingly been used within experimental cue reactivity research [33], relatively little work has examined whether these approaches can improve clinical outcomes in real-world treatment settings. VR-CET offers a potentially transformative extension of traditional CET approaches by enabling exposure to highly realistic, multisensory, and ecologically valid substance-use environments that closely resemble the situations in which cravings and relapse commonly occur [34]. Greater ecological validity may strengthen extinction learning and improve the transfer of treatment gains beyond the therapy setting [20,35]. Preliminary experimental and clinical studies have reported encouraging findings for VR-CET across a range of substance use disorders [15,16,36–39], yet rigorous evaluation within routine treatment services remains extremely limited. This study could contribute to improving the methodological quality of VR-CET research. Previous studies have often been limited by small sample sizes, lack of active control conditions, and a reliance on subjective outcomes. By incorporating a randomised design, an active VR control condition, physiological and subjective measures of cue reactivity, and assessments of implementation within routine services, the study will provide evidence regarding feasibility, acceptability, and trial procedures required for a future effectiveness trial.

The study also introduces a novel combination of immersive VR, wearable sensing technology, and JITA-EMA. Continuous physiological monitoring provides an opportunity to assess cue reactivity using objective biomarkers alongside traditional self-report measures [40]. Research has shown in healthcare that outcomes improve when routinely monitored [41]. If these physiological indicators prove sensitive to cocaine-related cue exposure and craving states, they could provide valuable for future research and treatment evaluation [42,43]. Furthermore, wearable-triggered JITA-EMA enables craving assessments to be delivered when physiological data suggest participants may be entering a high-risk state [44]. This could be an important step towards the future development of just-in-time adaptive interventions (JITAIs), in which support could be delivered in the moment when individuals are most vulnerable to craving or relapse [45].

Finally, the VR environments used in this study have been designed to maximise ecological validity by recreating realistic cocaine-related environments and social situations identified through lived-experience input and previous work [22]. Participants will be exposed to scenarios that mirror common real-world triggers, including drug purchasing, drug use, social interactions, and environmental contexts associated with cocaine consumption. This directly addresses a criticism of traditional cue exposure paradigms, namely that static laboratory cues may fail to address the complexity of real-world drug use triggers [46].

Taken together, this study addresses a significant gap in the evidence base by providing an evaluation of the feasibility and implementation potential of VR-CET within UK drug treatment services. If feasibility is demonstrated and subsequent efficacy trials prove successful, this programme of research could help shift addiction treatment from conventional laboratory-based cue exposure approaches towards a more sophisticated model that combines immersive technologies, such as VR, continuous physiological monitoring, real-time craving detection, and personalised intervention delivery. The findings will therefore be relevant in determining whether VR-CET can progress from an emerging experimental approach to a scalable, evidence-based intervention for substance use disorders.

### Limitations

Several limitations should be acknowledged. First, due to the nature of the VR intervention, neither participants nor intervention-delivery staff can be blinded to allocation; and, given the small research team, outcome assessors at the 8-week follow-up cannot be fully blinded. To mitigate differential expectancy, both arms use credible VR content, and treatment credibility will be assessed formally after Session 1. Second, the study relies partly on self-reported outcome measures (e.g., craving via CEQ-S, substance use via TLFB and EMA), which are subject to recall bias and social desirability effects. Third, as a feasibility trial with a sample of 60, the study is not powered to detect clinical efficacy, and any between-arm differences in clinical outcomes should be interpreted as indicative estimates only. Fourth, recruitment from specialist treatment-seeking samples may limit generalisability to cocaine users not engaged with services. Fifth, EMA and wearable adherence over 8 weeks may decline over time; patterns of missing data will be reported transparently. These limitations are inherent to the feasibility phase and will directly inform the design of a future definitive trial.

### Conclusion

This study aims to assess the feasibility of conducting this study on a larger scale, and any facilitating or barrier factors that should be considered in a larger study. If shown that VR-CET could be an effective treatment option for cocaine treatment, it will be important to consider future implementation within drug services as a novel approach, with the necessary further investigation.

## Abbreviations

CEQ-S: Craving Experience Questionnaire – Strength
CET: Cue Exposure Therapy
CGL: Change Grow Live
CI: Chief Investigator
CONSORT: Consolidated Standards of Reporting Trials
CUD: Cocaine Use Disorder
DMC: Data Monitoring Committee
EMA: Ecological Momentary Assessment
GAD-7: Generalised Anxiety Disorder
GCP: Good Clinical Practice
ISG: Independent Steering Group
JITA: Just In Time Adaptation
JITA-EMA: Just In Time Adaptation – Ecological Momentary Assessment
KCL: King’s College London
MPS: Multimodal Presence Scale
NHS: National Health Service
NIHR: National Institute for Health Care and Research
PHQ-8: Patient Health Questionnaire
PIS: Participant Information Sheet
PMG: Project Management Group
PPI: Participant and Public Involvement
PSS-10: Perceived Stress Scale
RADAR: Remote Assessment of Disease and Relapse
RCT: Randomised Controlled Trial
REC: Research Ethics Committee
SAE: Serious Adverse Event
SLaM: South London and Maudsley
SOP: Standard Operating Procedure
SUD: Substance Use Disorder
TAPS: Tobacco, Alcohol, Prescription medication, and other Substance use (TAPS) Tool
TAU: Treatment As Usual
TFA: Theoretical Framework of Acceptability
TLFB: Timeline Follow Back
UK: United Kingdom
VAS: Visual Analogue Scale
VR: Virtual Reality
VR-CET: Virtual Reality – Cue Exposure Therapy.
